# The Joint-Brain Axis: Insights From Rheumatoid Arthritis on the Crosstalk Between Chronic Peripheral Inflammation and the Brain

**DOI:** 10.3389/fimmu.2020.612104

**Published:** 2020-12-10

**Authors:** Patrick Süß, Tobias Rothe, Alana Hoffmann, Johannes C. M. Schlachetzki, Jürgen Winkler

**Affiliations:** ^1^ Department of Molecular Neurology, Friedrich-Alexander-University Erlangen-Nürnberg, University Hospital Erlangen, Erlangen, Germany; ^2^ Department of Neurology, Friedrich-Alexander-University Erlangen-Nürnberg, University Hospital Erlangen, Erlangen, Germany; ^3^ Department of Internal Medicine 3, Friedrich-Alexander-University Erlangen-Nürnberg, University Hospital Erlangen, Erlangen, Germany; ^4^ Department of Cellular and Molecular Medicine, University of California, San Diego, La Jolla, CA, United States

**Keywords:** rheumatoid arthritis, neurodegenenerative diseases, depression, blood-brain barrer, microglia, neuroinflammation

## Abstract

Rheumatoid arthritis (RA) is a chronic inflammatory disease characterized by erosive polyarthritis. Beyond joint pathology, RA is associated with neuropsychiatric comorbidity including depression, anxiety, and an increased risk to develop neurodegenerative diseases in later life. Studies investigating the central nervous system (CNS) in preclinical models of RA have leveraged the understanding of the intimate crosstalk between peripheral and central immune responses. This mini review summarizes the current knowledge of CNS comorbidity in RA patients and known underlying cellular mechanisms. We focus on the differential regulation of CNS myeloid and glial cells in different mouse models of RA reflecting different patterns of peripheral immune activation. Moreover, we address CNS responses to anti-inflammatory treatment in human RA patients and mice. Finally, to illustrate the bidirectional communication between the CNS and chronic peripheral inflammation, we present the current knowledge about the impact of the CNS on arthritis. A comprehensive understanding of the crosstalk between the CNS and chronic peripheral inflammation will help to identify RA patients at risk of developing CNS comorbidity, setting the path for future therapeutic approaches in both RA and neuropsychiatric diseases.

## Introduction

The central nervous system (CNS) has long been considered to be protected from circulatory inflammatory signals by the blood-brain barrier (BBB). However, an intimate crosstalk between chronic peripheral inflammation and the CNS is evidenced by a plethora of neurological and psychiatric sequelae associated with chronic inflammatory diseases like rheumatoid arthritis (RA).

RA is a systemic autoimmune disease characterized by synovial inflammation and deformation of joints and adjacent bones. The pathogenesis of RA is driven by a complex interplay between the adaptive immune system involving T-cells and autoantibodies as well as innate immune components like myeloid cells and pro-inflammatory cytokines (1–3). RA patients are highly predisposed to develop neuropsychiatric comorbidities. The prevalence of major depressive disorder in RA patients was estimated to be 16.8%, by far exceeding the general population (4, 5). Moreover, RA patients show higher levels of anxiety (6) and impaired cognitive performance (7, 8) compared to healthy individuals. Additionally, almost 40% of RA patients experience chronic pain, which is further linked to depression and anxiety (9). Interestingly, mid-life RA lead to an increased risk to develop dementia by 2.5-fold after a follow-up period of 21 years (10). However, epidemiological studies on the association between RA and individual neurodegenerative diseases like Alzheimer’s Disease (AD) and Parkinson’s Disease (PD) showed contradictory results (11–16). Nevertheless, RA therapeutics inhibiting pathogenetic pro-inflammatory cytokines like tumor necrosis factor (TNF) and interleukin-6 (IL-6) alleviate symptoms such as depression and anxiety (17, 18) and were linked to a reduced risk of future neurodegeneration (19) in RA patients.

While precise pathological mechanisms causing CNS involvement in RA are currently being investigated, most existing insights about the propagation of peripheral inflammation into the CNS and subsequent impairment of neural function are derived from animal models of acute infection by administration of lipopolysaccharide (LPS) or polyinosinic-polycytidylic acid (Poly(I:C)). In this context, peripheral inflammatory mediators can enter the CNS across the BBB or the choroid plexus, by the infiltration of blood-derived immune cells or the inflammatory activation of endothelial cells (20–23). Additionally, astrocytes and CNS-associated myeloid cells like parenchymal microglia, meningeal, perivascular and choroid plexus macrophages, acquire an inflammatory state. These changes are referred to as “neuroinflammation” and may ultimately be the link to neuropsychiatric symptoms by inducing neuronal damage, impaired adult hippocampal neurogenesis, and altered neurotransmitter signaling (20, 24–26).

Transferring these findings into the context of chronic peripheral inflammation is urgently needed as neuropsychiatric comorbidity in RA substantially contributes to disease burden and worsens therapeutic response and outcome (27, 28). However, research on this topic is hampered by the heterogeneity of present experimental models (29) and immunophenotypes observed in RA patients (30–32). The present review summarizes our current knowledge about inflammatory alterations and neuronal dysfunction in the brain of RA patients and rodent models. Vice versa, we will also discuss how the CNS is able to modulate the course of peripheral arthritis. Additionally, we aim to highlight open questions and future research strategies to better decipher and treat neuropsychiatric vulnerability in chronic peripheral inflammation.

## Genetic Links Between Chronic Inflammation and Neurodegeneration

The clinical CNS involvement of some patients with RA has raised the question of a shared genetic predisposition for RA and neurological or psychiatric diseases. A particular focus was aimed towards neurodegenerative diseases, since the immune system is increasingly being acknowledged as an important driver of pathogenesis. Recently, a comparison of genome-wide association studies (GWAS) on neurodegenerative and chronic immune-mediated diseases revealed 15 single-nucleotide polymorphisms (SNPs) jointly associated with RA and frontotemporal dementia (FTD). Interestingly, the majority of those SNPs were located in the human leukocyte antigen (HLA) region on chromosome 6 (33). This region encodes a set of gene products essential for self- and non-self-antigen presentation and immune function both in the periphery and the CNS. The dense and overlapping organization of HLA-genes on chromosome 6 did not allow the identification of individual genes accounting for the shared risk between FTD and RA. Shared disease-associated SNPs related to immune function were also identified for RA and PD (34), amyotrophic lateral sclerosis (ALS) and progressive supranuclear palsy (PSP) (33). Yokoyama et al. identified few SNPs jointly associated with AD and different immune diseases including RA (35). In contrast, Mendelian Randomization studies showed no positive correlation between known genetic risk factors for RA and incidence of AD (15, 36) or vice versa (37). Interestingly, the polygenic risk for RA integrating many known predisposing SNPs was correlated with lower cognitive performance in healthy adolescents (38). Felsky et al. demonstrated a correlation between polygenic risk for RA and microglial density in the brain of elderly individuals (39). Again, this correlation was substantially driven by genetic changes located in the HLA region on chromosome 6 (39). Taken together, HLA-associated polymorphisms and immune-related genetic risk factors for RA may contribute to comorbid cognitive impairment and neurodegenerative diseases.

## Routes Conveying Chronic Peripheral Inflammation to the Brain

**Figure 1:**
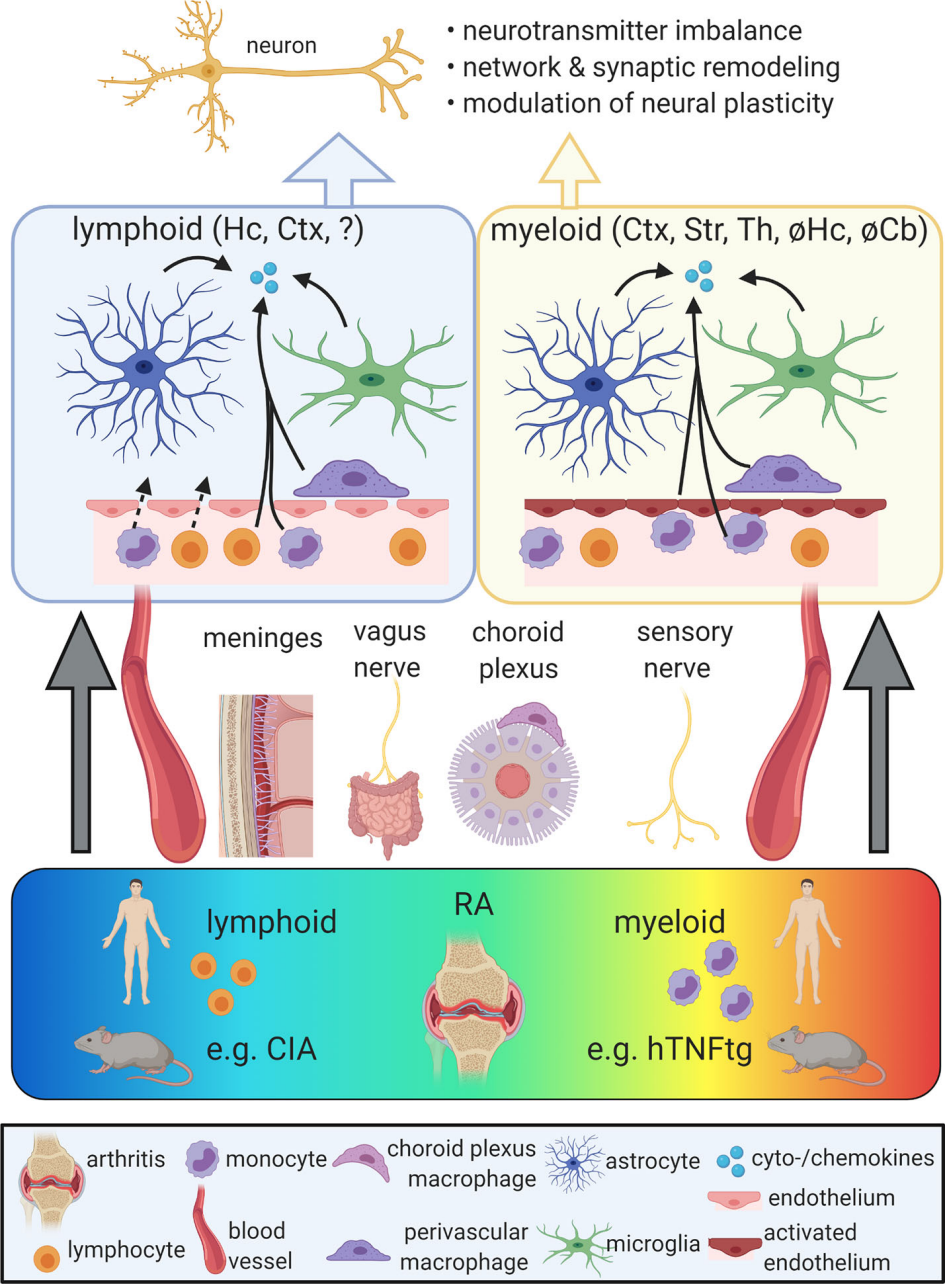
Propagation of chronic peripheral inflammation into the central nervous system (CNS). Rheumatoid arthritis (RA) comprises a spectrum of different peripheral immunophenotypes, including a lymphoid subtype driven by adaptive immune activation and a myeloid subtype characterized by the activation of myeloid cells. While the lymphoid subtype is represented by the mouse model of collagen-induced arthritis (CIA), the human TNF-α transgenic (hTNFtg) mouse model mimicks key aspects of the myeloid form of RA. Peripheral inflammation can reach the CNS *via* different gateways, including the vagus nerve, the somatosensory nervous system, the meninges, the choroid plexus and the bloodstream. In both lymphoid and myeloid models of RA, the activation of microglia, astrocytes, and perivascular macrophages as well as increased levels of pro-inflammatory cytokines and chemokines were described. While in CIA, these alterations were mainly observed in the cortex (Ctx) and hippocampus (Hc), hTNFtg mice show a distinct regional distribution of neuroinflammation including the Ctx, striatum (Str), and thalamus (Th), but sparing the Hc and the cerebellum (Cb). The blood-brain barrier (BBB) was proposed to be disrupted in lymphoid models, potentially allowing the influx of blood-derived immune cells. In myeloid models, BBB integrity appears maintained, but endothelia display an activated signature and may contribute to the secretion of cytokines and chemokines. Neuroinflammation in RA models was linked with impaired neuronal function due to altered neurotransmitter metabolism and neural plasticity as well as synaptic and network refinement. Ultimately, these changes may cause neuropsychiatric symptoms. So far, behavioral phenotypes were mainly found in lymphoid models of RA and are limited in the myeloid subtype. Figure created with BioRender (https://biorender.com).

To induce neuroinflammation in the context of arthritis, peripheral inflammatory signals must enter into the CNS. This is achieved by several routes (**Figure 1**). First, circulatory pro-inflammatory cytokines are able to enter the brain by volume diffusion in circumventricular organs (CVOs), neuroanatomical sites of increased BBB permeability located around the third and fourth ventricles (20). Besides, studies based on LPS-induced acute peripheral inflammation proposed the entrance of cytokines into the brain by active transport or tight junction damage (40, 41). In the context of RA, disruption of the BBB was observed in a collagen-induced arthritis (CIA) model (42, 43). This model is based on immunization against collagen-II and strongly driven by T-cell-dependent mechanisms. Correspondingly, brain homogenates of CIA-induced mice showed an increased cell population expressing monocyte markers C-C motif chemokine receptor 2 (Ccr2) and Ly6c (44). This finding might indicate blood-derived myeloid cell infiltration into the brain parenchyma, although perivascular or meningeal localization of the detected cells was not excluded. In contrast to CIA, BBB tight junctions remained intact in mice overexpressing human TNF in the periphery (hTNFtg) (45), a model characterized by a profound myeloid cell activation without T-cell involvement (46, 47). In line with BBB integrity in this model, single cell RNA-seq of cortical myeloid cells showed no increase in the number of blood-derived monocytes or granulocytes (45). In summary, findings on BBB disruption differ between RA animal models and evidence for CNS infiltration of peripheral myeloid cells in RA is limited. The role of T cells in the CNS involvement of RA is largely unaddressed.

Peripheral inflammation may also induce non-disruptive alterations of the BBB, including an upregulation of leukocyte adhesion markers and the secretion of inflammatory mediators by endothelial cells (48). In hTNFtg mice, intercellular adhesion molecule 1 (Icam-1) and vascular cell adhesion molecule 1 (Vcam-1) are induced in distinct brain regions, indicating endothelial activation without tight junction leakage (45). Interestingly, endothelial Vcam-1 was recently described to mediate neuroinflammation and cognitive impairment during aging. This process was accompanied by intravascular adhesion, but not parenchymal infiltration of circulatory leukocytes (49). In Complete Freud’s Adjuvant-induced arthritis, endothelia contribute to vessel-associated micro- and astrogliosis and subsequent hyperalgesia by vascular endothelial growth factor 2 (Vegf2)-dependent upregulation of Icam1 in the spinal cord (50). Hence, BBB endothelial cells may act as an active mediator of neuroinflammation rather than a passive barrier in the context of RA.

Moreover, chronic peripheral inflammation is propagated into the CNS by direct neuronal routes. Pro-inflammatory cytokines like IL-1β, IL-6, and IL-17 were shown to activate peripheral nociceptive afferents (51). These neuronal afferents are involved in the pathogenesis of arthritis, but also signal inflammatory cues to the CNS. Accordingly, Hess et al. demonstrated a profoundly increased pain response in the brains of hTNFtg mice and RA patients by functional magnetic resonance imaging (fMRI), which was reversible upon inhibition of peripheral TNF (52). This modulation of CNS activity preceded the histopathological amelioration of arthritis (52). Apart from the somato-afferent nervous system, the vagus nerve may display a second neuronal afferent route linking chronic peripheral inflammation and the CNS. The vagal nerve is involved in the generation of behavioral responses to LPS administration (53) and activated by TNF and IL-1β in cytokine-specific electrophysiological patterns (54). Future experiments transferring these findings into the context of arthritis are needed.

Finally, further interfaces between the peripheral immune system and the CNS, including the choroid plexus, the meninges and the glymphatic system have hardly been studied in chronic peripheral inflammation and might provide further insights into the involvement of the CNS in RA. Moreover, gut microbiota can modulate microglia and brain function (55, 56). As gut dysbiosis was shown in RA (57), the gut-brain axis might as well contribute to RA-associated neuropsychiatric comorbidity.

## Neuroinflammation in RA

After receiving inflammatory signals from the periphery, CNS-resident cells, particularly microglia and astrocytes, are able to acquire an activated phenotype and maintain a neuroinflammatory state. Recently, we characterized myeloid cells in hTNFtg mice by histology, flow cytometry, and scRNA-seq. We detected a strong microglial activation signature with a downregulation of homeostatic markers like transmembrane protein 119 (Tmem119), P2ry12, and Fc receptor-like S (Fcrls) accompanied by the upregulation of CD45, sialic acid-binding immunoglobulin-type lectin 1 (Siglec-1), several complement factors, and chemokines as well as genes linked to lysosomal function (45). Importantly, this microglial response was restricted to defined brain regions, including the cortex, striatum, and thalamus, and reversed by inhibition of peripheral human TNF using infliximab, a clinically used compound for the treatment of RA (45). In the cerebellum and hippocampus of hTNFtg mice, there was little, if any, inflammatory response. This regional vulnerability of the CNS may be linked to locally confined endothelial activation (45). The low susceptibility of the hippocampus, a region involved in the pathophysiology of depression and memory disorders, may explain the absence of depressive-like symptoms in hTNFtg mice (58). When these mice were crossed with the 5XFAD model of AD, decreased amyloid plaque load was detected. However, the authors also suggested impaired synaptic integrity, which might be due to an unselectively boosted phagocytosis of activated myeloid cells (59). Behavioral analyses are needed to answer the question if TNF-driven arthritis overall ameliorates or worsens AD-like phenotypes in 5XFAD mice.

In contrast to microglia, the response of astrocytes is poorly characterized in TNF-driven arthritis and only based on the expression of glial fibrillary acidic protein (Gfap). While in hTNFtg mice, reactive astrogliosis was concluded by increased Gfap staining intensity in the cortex (59), arthritis driven by overexpression of murine TNF was associated with activation of microglia, but not astrocytes (60).

In contrast to TNF-driven arthritis, lymphoid cell-based models of RA like CIA or antigen-induced arthritis (AIA) were less extensively investigated regarding neuroinflammation, mainly focussing on the hippocampus and cortex. Several studies reported an elevated expression of TNF, IL-1β, or IL-6 in the hippocampus and cortex of arthritic mice (43, 44, 61, 62). Moreover, microglial density and expression of the phagocytosis marker CD68 were increased in the hippocampus during CIA (43, 61). Increased phagocytosis was corroborated by a decrease of amyloid or tau pathology, when CIA was induced in AD mouse models (43, 44). However, analyses of neuronal integrity and behavior were not performed in these studies. Besides activation of microglia, CIA led to increased numbers of Gfap^+^ astrocytes in the hippocampus (61). Hippocampal inflammation in CIA and AIA was associated with depressive-like behavior (61, 62).

Together, these data show neuroinflammation in immunization-based RA models. Interestingly, hippocampal immune response in these models indicates a different regional pattern of neuroinflammation compared to myeloid cell-based, TNF-driven arthritis. It is therefore tempting to speculate, that the peripheral immunophenotype during chronic inflammation navigates regional neuroinflammation. Importantly, several recent transcriptomic studies point out the existence of different immunophenotypes in RA patients (30–32). This includes phenotypes with lymphoid-based and myeloid-based inflammation (30, 32), which reflects the pathophysiological hallmarks of CIA and the hTNFtg model, respectively. Thus, a comparison of BBB alterations, neuroinflammation, and neuropsychiatric comorbidity between these different subtypes of RA patients might help to understand predisposing factors for neurological or psychiatric comorbidity.

In RA patients, neuroinflammation has hardly been examined. Analyses of human *post mortem* brain tissue indicated microglial activation evident by downregulation of the homeostatic marker P2RY12 in the cortex, but not the cerebellum of RA patients. This finding matches with the regional CNS immune response previously observed in hTNFtg mice (45). The cerebrospinal fluid of RA patients contained increased levels of IL-1β compared to multiple sclerosis (MS) patients and healthy controls (63). Moreover, longitudinal proteomic analyses of CSF samples derived from seven RA patients prior to and during infliximab treatment identified a set of immune-associated markers including complement factor B, which were reduced by TNF blockade (64). Future studies are needed to translate further key aspects of neuroinflammation detected in rodent arthritis models into human diseased conditions.

## Structural Alterations and Neuronal Dysfunction in RA

To eventually result in neuropsychiatric symptoms, neuroinflammation is associated with impaired neuronal function. In this regard, several mechanisms have been proposed: alterations in neurotransmitter signaling, dynamic modulation of dendritic spines and neuronal networks, and impaired adult hippocampal neurogenesis.

First, chronic peripheral inflammation was reported to affect glutamatergic and serotonergic signaling. In a rat model of 2,4,6-trinitrobenzenesulfonic acid (TNBS)-induced colitis, hippocampal inflammation triggered altered glutamatergic signal transduction, which was reversible upon anti-inflammatory treatment (65). In the pathogenesis of depression, serotonin, a derivative of the essential amino acid tryptophan, plays a key role. Krishnadas et al. observed that serotonin transporter activity in the brainstem assessed by nuclear imaging positively correlated with serum levels of TNF and depressive symptoms (66). In patients with psoriatic arthritis, treatment with the TNF antagonist etanercept for 6–8 weeks significantly reduced serotonin transporter activity, thereby increasing serotonin availability in the synaptic cleft (66). Collectively, chronic peripheral inflammation in RA may induce neurotransmitter dysregulation in the CNS. As neurotransmitter metabolism and recycling is a major homeostatic function of astrocytes, a better understanding of astrocytic modulation during RA-induced neuroinflammation may also provide further insights into the role of different neurotransmitters in arthritis-associated neuropsychiatric symptoms.

Secondly, changes in neuronal structure subsequent to chronic peripheral inflammation may account for neuropsychiatric symptoms. In acute inflammation induced by Poly(I:C), Garré et al. observed increased loss of cortical dendritic spines functionally resulting in learning deficits. Of note, these changes were independent of microglia, but orchestrated by peripheral C-X3-C motif chemokine receptor 1 (Cx3cr1)-expressing monocytes and TNF (23). As TNF was linked to depressive symptoms in AIA (62), it is of great interest to examine dendritic spine dynamics in RA mouse models. Besides remodeling dendritic spines, chronic peripheral inflammation may induce complex structural network rearrangements in the brain. Schrepf et al. demonstrated rewiring of the brain connectome by multimodal brain MRI in RA patients. In particular, the inferior parietal lobe and the medial prefrontal cortex were more strongly involved in several brain networks compared to healthy controls (67).

Adult hippocampal neurogenesis, the generation of new neurons in the dentate gyrus throughout lifetime, is a physiological process contributing to learning, memory, pattern separation and emotions. Adult hippocampal neurogenesis is impaired in models of neurodegenerative diseases (68) and depression (69), but is also affected by local and systemic inflammation (24, 70). It has been investigated in several RA models. Interestingly, in rodent models mimicking the lymphoid subtype of RA, divergent findings were obtained. In AIA, a slight increase in the proliferation of neural progenitor cells and the number of surviving newly generated neurons were described (71, 72). In contrast, Andersson et al. recently showed reduced adult hippocampal neurogenesis and smaller hippocampal volume in mice with CIA (61). Impaired adult hippocampal neurogenesis was mediated by inflammation-induced insulin-like growth factor 1 receptor (IGF1R) signaling. Interestingly, small hippocampal volume in female RA patients correlated with more severe pain and reduced levels of serum insulin-like growth factor 1 (IGF1) (61). Moreover, high IGF1R expression in leukocytes of RA patients significantly correlated with symptoms of anxiety (61). Of note, the study was restricted to female RA patients. It will be interesting to study the role of IGF1/IGF1R in male RA patients, as the role of gender in CNS involvement during RA is hardly investigated. Taken together, the IGF1/IGF1R-axis might therefore serve as a biomarker for some neuropsychiatric symptoms in RA patients. In contrast to lymphoid-based models, a stepwise characterization of adult hippocampal neurogenesis in the myeloid-like hTNFtg model revealed no difference compared to wt controls (58). This is in line with the observed resilience of the hippocampus to neuroinflammation in this model (45, 58).

In summary, chronic peripheral inflammation during arthritis is propagated to the CNS and subsequently causes neuropsychiatric symptoms by affecting neurotransmitter metabolism, dendritic spine and neuronal network dynamics adult hippocampal neurogenesis. It is important to note, that these changes appear to depend on the particular peripheral immunophenotype.

## CNS Modulation of Arthritis

After highlighting the modulation of the CNS by RA, it is noteworthy that recent studies propose effects of the CNS on the severity and progression of arthritis, which are mainly mediated by the autonomic nervous system. These findings illustrate the reciprocal interaction between CNS pathology and RA and are in line with the clinical observation that depression is frequent in RA, but also predisposes for RA development (73).

To date, different mechanisms of systemic immune modulation by the CNS have been revealed. As a major efferent route mediating immune suppression by the CNS, the vagal nerve controls the production of TNF and other pro-inflammatory cytokines (74). This effect is mediated *via* the splenic nerve, which directly activates β-adrenergic receptors on splenic CD4^+^ T cells expressing choline acetyltransferase (ChAT). ChAT^+^CD4^+^ T cells in turn suppress cytokine production in other immune cells by cholinergic signaling *via* the α7 nicotinic acetylcholine receptor (75, 76). The therapeutic potential of vagal nerve stimulation has been demonstrated in a rat model of CIA (77) and a small group of RA patients, leading to attenuated cytokine production and decreased disease activity scores (78). Moreover, direct stimulation of splenic nerve terminals by ultrasound altered gene expression profiles of B and T cells and alleviated arthritis in the K/BxN serum transfer model, which is mediated by antibodies against glucose-6-phosphate isomerase (79). To date, it is hardly understood, how this modulation of chronic peripheral inflammation *via* the autonomic nervous system is orchestrated by central brain regions. Two recent studies showed that central stimulation of the locus coeruleus and the parietal cortex dampened zymosan-induced arthritis in rats *via* sympathetic adrenergic signaling to affected joints (80, 81). Interestingly, both brain regions are activated by afferent vagal stimulation (80, 81).

Efferent modulation of peripheral inflammation by the CNS raises the question, how neurological diseases influence the risk and severity of arthritis. Lang et al. observed that neurodegeneration in transgenic mice expressing the human tau P301S mutant (P301S mice) was linked to higher induction rates and earlier onset of CIA (44). As the number of ChAT^+^ T cells in the spleen was not different in P301S mice (44), CNS modulation of arthritis might be independent of the vagus efferent pathway.

Post-stroke immunosuppression is a transient condition of mitigated immune function and increased vulnerability towards infection following cerebral ischemia. This phenomenon was recently linked to the suppression of peripheral natural killer (NK) cells by catecholaminergic and glucocorticoid signaling *via* the sympathetic nervous system and the hypothalamo-pituitary-adrenal axis (82). Intriguingly, post-stroke immunosuppression alleviated K/BxN serum transfer-induced arthritis in mice during early disease stages (83). Collectively, these data suggest that certain CNS regions may on the one hand be a target, but on the other hand also a modulator of chronic peripheral inflammation in the context of RA.

## Conclusion

Besides joint pathology, patients with RA frequently show highly relevant comorbidities involving the CNS resulting in aggravated therapeutic response and outcome. Neuropsychiatric and neurodegenerative disorders associated with RA are thought to be at least partially linked to neuroinflammation targeting specific brain regions. However, the extent of neuroinflammation and how much it contributes to CNS comorbidities in RA is still unclear. More studies targeting the brain myeloid cell compartment in RA and other peripheral immune diseases like ulcerative colitis would shed more light on the pathogenesis of CNS comorbidities. In this regard, the amelioration by TNF inhibitors like infliximab strongly implies an RA-linked neuroinflammatory response different from MS, which is aggravated or triggered by TNF inhibition (84). The CNS myeloid cell activation pattern in RA was reported to be similar to the disease-associated microglia (DAM) profile observed in neurodegenerative diseases (45, 85), an activation state distinct from the pattern observed in the experimental autoimmune encephalomyelitis model of MS (86). One exciting line of research would be to explore a potential link of complement-dependent synaptic degeneration as postulated in AD (87, 88) in the context of RA.

The heterogeneity in BBB modulation, myeloid cell activation, regional neuroinflammation, and adult hippocampal neurogenesis observed in different RA mouse models will have to be related to the distinct immunophenotypes in RA patients. A major goal is the identification of novel biomarkers defining RA patients at risk of CNS involvement to enable an early interdisciplinary treatment. Vice versa, such biomarkers may help to predict the risk of future arthritis in patients with pre-existing neuropsychiatric diseases, such as depression.

Finally, the bidirectional interaction between chronic peripheral inflammation and the brain will enable innovative treatment approaches for systemic inflammatory, neurological, and psychiatric diseases.

## Author Contributions

PS and TR drafted the manuscript. PS designed the figure. PS, AH, JS, and JW discussed and revised the manuscript. All authors contributed to the article and approved the submitted version.

## Conflict of Interest

The authors declare that the research was conducted in the absence of any commercial or financial relationships that could be construed as a potential conflict of interest.

The handling editor declared a past co-authorship with one of the authors, PS.
